# Dark zone of the Greenland Ice Sheet controlled by distributed biologically-active impurities

**DOI:** 10.1038/s41467-018-03353-2

**Published:** 2018-03-14

**Authors:** Jonathan C. Ryan, Alun Hubbard, Marek Stibal, Tristram D. Irvine-Fynn, Joseph Cook, Laurence C. Smith, Karen Cameron, Jason Box

**Affiliations:** 1 Centre for Glaciology, Department of Geography and Earth Sciences, Aberystwyth University, Aberystwyth, SY23 3DB UK; 2 Department of Geography, University of California, Los Angeles, Los Angeles, CA 90095 USA; 3 Institute at Brown for Environment and Society, Brown University, Providence, RI 02906 USA; 4 Centre for Arctic Gas Hydrate, Environment and Climate, Department of Geology, University of Tromsø, 9037 Tromsø, Norway; 5 Department of Ecology, Faculty of Science, Charles University, 12844 Prague, Czech Republic; 6 Department of Geochemistry, Geological Survey of Denmark and Greenland, 1350 Copenhagen, Denmark; 7 Department of Geography, University of Sheffield, Sheffield, S10 2TN UK; 8 Institute of Biological, Environmental and Rural Sciences, Aberystwyth University, Aberystwyth, SY23 3DB UK; 9 Department of Glaciology and Climate, Geological Survey of Denmark and Greenland, 1350 Copenhagen, Denmark

## Abstract

Albedo—a primary control on surface melt—varies considerably across the Greenland Ice Sheet yet the specific surface types that comprise its dark zone remain unquantified. Here we use UAV imagery to attribute seven distinct surface types to observed albedo along a 25 km transect dissecting the western, ablating sector of the ice sheet. Our results demonstrate that distributed surface impurities—an admixture of dust, black carbon and pigmented algae—explain 73% of the observed spatial variability in albedo and are responsible for the dark zone itself. Crevassing and supraglacial water also drive albedo reduction but due to their limited extent, explain just 12 and 15% of the observed variability respectively. Cryoconite, concentrated in large holes or fluvial deposits, is the darkest surface type but accounts for <1% of the area and has minimal impact. We propose that the ongoing emergence and dispersal of distributed impurities, amplified by enhanced ablation and biological activity, will drive future expansion of Greenland's dark zone.

## Abstract (web summary)

The surface types that comprise the dark zone of the Greenland Ice Sheet, an area of bare ice with low albedo, are unknown. Here, the authors use UAV imagery to show that, during the melt-season, biologically active surface impurities are responsible for spatial albedo patterns and the dark zone itself.

## Introduction

The Greenland Ice Sheet has become the largest cryospheric contributor to global sea-level rise predominantly through increased surface melt and runoff, which accounts for over half of its mass loss since 1991^1–4^. The dominant energy source for snow and ice melt is direct solar shortwave radiation, the absorption and reflection of which is predominantly modulated by surface albedo^5–7^. Accurately constraining spatiotemporal patterns of albedo across the ice sheet is hence fundamental to understanding and predicting surface melt and runoff along with their impact on ice sheet flow dynamics and sea-level rise. A conspicuous feature of Greenland’s ablation area is its dark zone, an area of bare ice with particularly low albedo that appears across the west and southwest sectors of the ice sheet each summer^8–10^. At the Arctic Circle, in the vicinity of the Kangerlussuaq (K-) sector, the dark zone extends between 20 and 75 km from the land-terminating margin where Moderate Resolution Imaging Spectroradiometer (MODIS) data indicate a regional albedo minimum of ~0.34^8^. From 2000 to 2012, the spatial extent of the dark zone increased by 12% but also exhibited considerable interannual variability^11,12^. The extent of the dark zone is weakly positively correlated with air temperature and negatively correlated with solar radiation during June, July and August (JJA)^11,12^. This suggests that the ongoing albedo decrease observed during the melt season is not simply driven by melting of the winter snowpack to reveal the darker bare ice surface beneath, but, following exposure, there are changes in the nature of the bare ice itself^11,12^. However, the surface characteristics of the dark zone remain unquantified because the spatial resolution of satellite imagery is insufficient to fully resolve the specific surface types that comprise it, and how these surfaces evolve through time, distinguishing the dark zone from brighter ice surfaces adjacent to it.

Previous field-based, in situ observations indicate that western Greenland’s ablation zone is characterized by highly variable non-ice constituents and surface structures^8,13–15^. These include features such as crevasses, fractures and foliations^16,17^; supraglacial hydrological features, including streams, rivers, ponds and lakes^18,19^; snow patches and fracture cornices; cryoconite, concentrated in holes or in supraglacial fluvial deposits^20,21^; microbes and their humic by-products^22–24^; mineral dust and aerosols from outcropping or contemporary aeolian deposition including black carbon from wildfires^10,25,26^ and other aerosols. While the highest resolution optical satellite imagery currently available has facilitated the examination of crevasse fields^16^ and surficial hydrology^19^, a quantitative assessment of the specific ice surface types that comprise the dark zone, and how they combine to yield observed albedo patterns across the ablation zone of the ice sheet, has yet to be made.

**Fig. 1:**
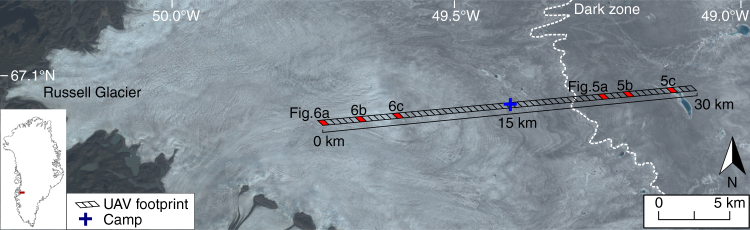
Overview map showing location of UAV survey transect. The background is a Landsat 8 Operational Land Imager (OLI) true colour image of the Kangerlussuaq sector of the Greenland Ice Sheet from 6 August 2014. The transect was divided into sixty 0.25 km^2^ segments for comparison with the MODIS albedo product, MOD10A1. High-resolution aerial imagery and surface classification of six segments (coloured red) are shown in Figs. 5 and 6

**Fig. 2:**
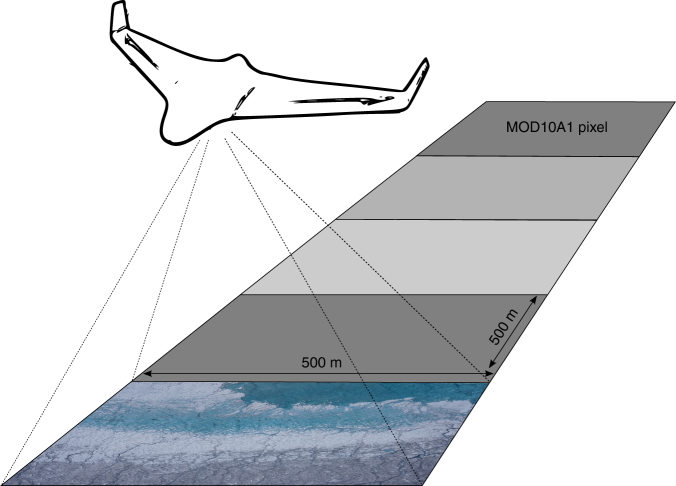
Schematic summarizing the aims of the study. Aerial digital imagery are used to characterize the surface types that are found in the ablation zone and assess their impact on mesoscale spatial albedo patterns as represented by MODIS

Here, we utilize high-resolution (15 cm pixel size) imagery acquired from an unmanned aerial vehicle (UAV) to characterize the specific ice surfaces across the dark zone and determine their impact on the mesoscale (1–10 km) albedo distribution during peak melt season, as represented by the MODIS albedo product, MOD10A1. On 8 August 2014, a fixed-wing UAV equipped with a digital camera and upward and downward facing pyranometers was deployed from a field camp based in the vicinity of the K-transect, S6 automated weather station (AWS) on a 25 km east-west transect dissecting the dark zone (Fig. 1). Seven distinct surface types were visually identified on the ground by an expert and automatically classified based on their reflectance and roughness properties. The survey transect was divided into sixty 500 × 500 m segments, co-located to the footprints of corresponding MODIS pixels, and the fractional area of each surface type in each segment was determined using a supervised k-nearest neighbours (k-NN) classification (see Methods section: Surface Classification for more information) (Figs. 1, 2). Finally, the mean albedo of each surface type was derived from the digital imagery and the relative contribution of different surface types to mesoscale albedo variability (defined by MOD10A1 pixels) was calculated using principal component regression (PCR).

## Results

### Surface type variation along the transect

**Fig. 3:**
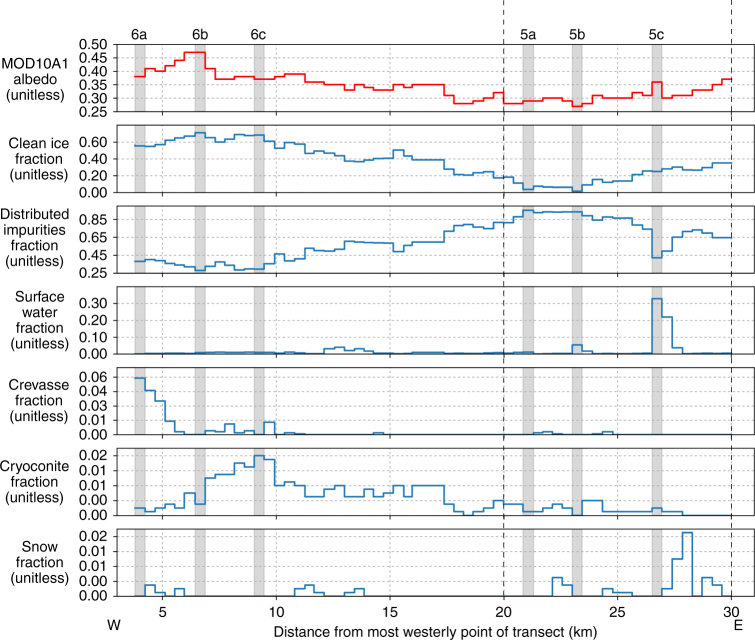
Variation of albedo and the fractional area of each surface type across the UAV transect. The albedo and fractional areas derived from MOD10A1 and the UAV imagery, respectively, on 8 August 2014. The *x* axis is displayed in Fig. 1. The results of the classification for six segments, highlighted by the vertical grey bars, are shown in Figs. 5 and 6

**Fig. 4:**
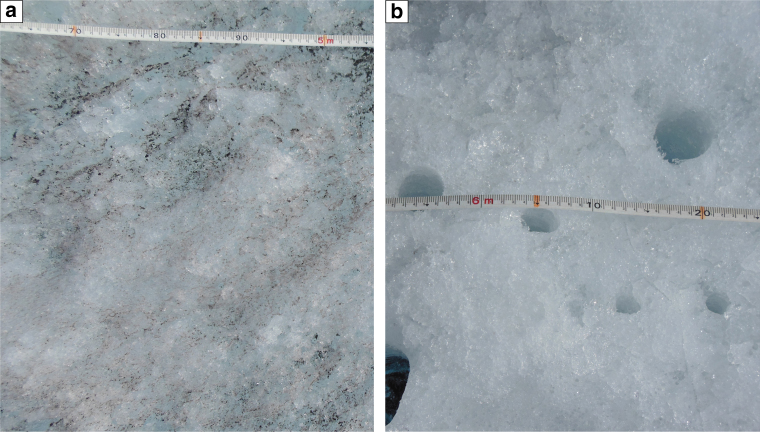
Photograph showing close-up of bare ice found in the ablation zone. The photographs were taken near the field camp located close to the S6 automated weather station at ~1000 m a.s.l. (Fig. 1). **a** Ice containing distributed impurities on the surface and **b** clean ice with cryoconite holes

**Fig. 5:**
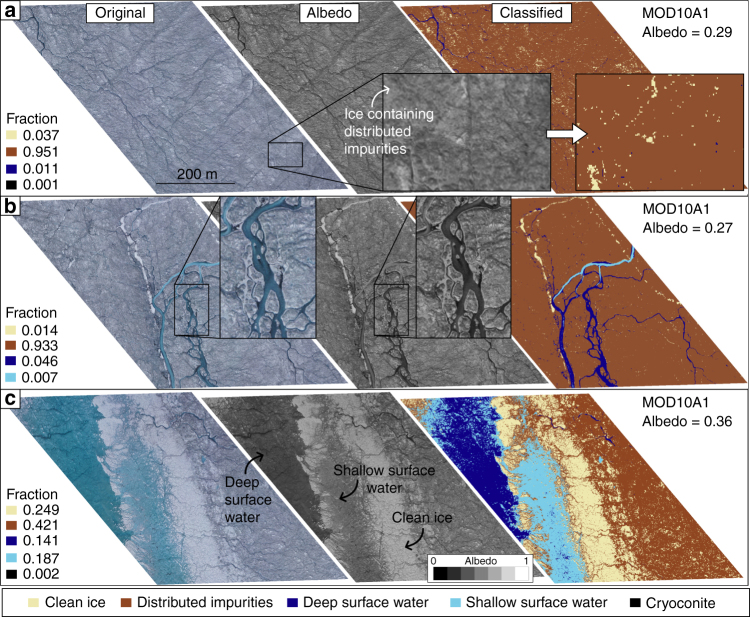
RGB digital image, albedo map and classification of surface types in three MOD10A1 pixels. The albedo maps were derived from the digital images (Methods). The locations of the segments along the UAV transect are shown in Fig. 1. **a** Segment characterized by mostly ice containing uniformly distributed impurities. **b** Segment characterized by similar ice surface to **a** but with a larger fraction of channelized surface melt-water. **c** Segment dominated by a supraglacial lake with a previous shore consisting of clean ice

Analysis of all UAV imagery allowed us to visually identify and automatically classify seven distinct surface types across the survey transect: (i) clean ice, (ii) ice containing uniformly distributed impurities, (iii) deep water, (iv) shallow water, (v) cryoconite either in holes or fluvial deposits, (vi) crevasses and (vii) snow (Fig. 3). Distinction between clean ice and ice containing uniformly distributed impurities was guided by qualitative assessment of 112 oblique and nadir photographs taken from the ground (<1 cm pixel footprint) at specific study sites around the field camp (Fig. 1). These images confirm that distributed impurities across the ice surface are responsible for bare ice albedo variability at the local scale (1–10 m) (Fig. 4). In order to upscale and understand the impact of these surface impurities on the mesoscale albedo distribution of the ablation zone, we divided bare ice into two categories: clean ice, with very low impurity concentrations, and ice containing some or an abundance of impurities. It is apparent that additional categories could be defined for bare ice given sufficiently high pixel resolution, but for the purpose of this study, and considering the spectral limitations of the onboard camera, we do not attempt to. We note that it would be a fruitful direction with multi- and hyper-spectral sensor payloads. Clean ice has 57.2% aerial coverage in the lower, western half of the survey transect between 0 and 17 km (Fig. 3). In the eastern half (17–27 km), clean ice coverage is lower at 23.0%. Ice containing uniformly distributed impurities (Fig. 5a) varies inversely to clean ice, with a higher fraction in the eastern half (74.5%) compared to the western half of the transect (40.0%) (Fig. 3).

**Fig. 6:**
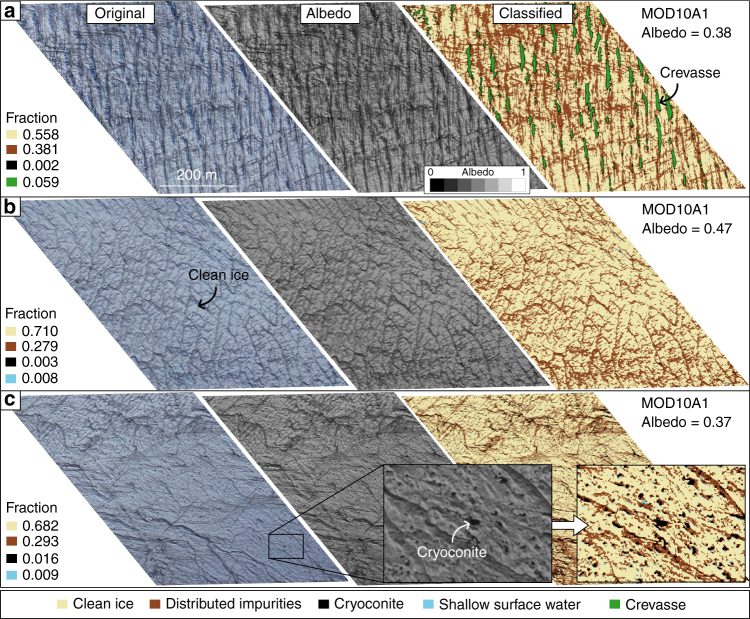
RGB digital image, albedo map and classification of surface types in three more MOD10A1 pixels. The albedo maps were derived from the digital images (Methods). The locations of the segments along the UAV transect are shown in Fig. 1. **a** Segment containing a high fraction of crevasses. **b** Segment characterized by a much lower relief surface and no crevasses. **c** Segment characterized by clean ice and numerous cryoconite holes

Cryoconite is commonly found in holes but also in fluvial deposits near supraglacial streams and lakes (Fig. 6c). In this study, cryoconite is distinguished from ice containing distributed impurities by its very low albedo, which is indicative of concentrated, rather than distributed, impurities. Cryoconite has a maximum aerial coverage of 1.6% at 8 km from the western end of the transect and a mean coverage of 0.6% across the entire transect (Fig. 3). It is possible that we underestimate the fractional area of small cryoconite holes due to the limited, 15 cm pixel resolution of our UAV imagery. However, we note that smaller cryoconite holes (<15 cm) would also be hidden from virtually all aerial and satellite imagery obtained at low solar elevation angles. Furthermore, cryoconite hole depths tend to equilibrate as the melt season progresses, due to their low albedo and preferential radiative absorption in comparison to brighter ice surfaces surrounding them^20,21^. Coincident field measurements, made during UAV image acquisition, indicate that the cryoconite holes observed in our study were well developed. The implication is that once they have attained equilibrium depth, they cease to absorb additional energy (which would make them deeper) compared to surrounding ice and hence are effectively neutralized from the effects of incoming solar radiation. For these reasons, we argue that smaller cryoconite holes had a minimal net impact on MODIS-derived albedo compared to the ice surface surrounding them.

At 23 km (all distances refer from the western start point of the UAV transect), a braided meltwater channel network with a fractional area of 5.3%, intersects the transect (Figs. 1 and 5b), but otherwise, surface water comprises only 1.9% of the survey area (Fig. 3). These results are consistent with Smith et al.^19^ who found that surface water accounted for 1.4% of a ~5000 km^2^ bare ice area in the K-sector of the ice sheet. At 28 km, a small, 0.83 km^2^ supraglacial lake covers 32.8% of the segment (Fig. 5c). Crevasse density is highest on the western flank between 2 and 5 km and attains maximum coverage of 5.9% at 2 km (Figs. 3, 6a). Up-glacier of 10 km, crevasses are almost entirely absent (Fig. 6b). Remnant snow patches, which persist within ice fractures and supraglacial channel incisions, attain a maximum coverage of 1.7% with a mean aerial coverage of only 0.1%, at this time of year.

### Relationship between surface types and albedo patterns

The mesoscale albedo distribution, determined from MOD10A1 data, exhibits considerable variability along the survey transect with values between 0.27 and 0.47 (Figs. 1, 3). The dark zone, which is approximately located between 18 and 27 km along the survey transect, has a mean albedo of 0.29 and is characterized by distinct and conspicuous banding that specifically relate to foliation structures apparent in Landsat 8 imagery (Fig. 1). Between 80 and 95% of the dark zone is classified as ice containing uniformly distributed impurities (mean albedo (*α*) = 0.27), with the remaining 5–20% consisting of predominately clean ice (mean *α* = 0.55) (Fig. 5a). Application of PCR reveals that the fractional area of ice containing uniformly distributed impurities explains 73% of the observed mesoscale albedo variability. Although not the darkest surface type observed, distributed impurities dominate the mesoscale albedo signal due to their extensive coverage and large variations in their fractional area across the survey transect. Distributed impurities have been attributed to the outcropping of aeolian dust deposited during the early Holocene^9,10,27^ and/or pigmented surface algal blooms and associated humic material^22–24^.

Locally, defined at the scale of a single MODIS pixel, supraglacial water, contained in both lakes and channels, has a distinct impact on albedo due to its low albedo (*α* = 0.19–0.26). The segment of the transect that corresponds to a large braided channel network at 23 km has an albedo of 0.28 (Fig. 5b). This is ~0.02 lower than the surrounding segments with <1% surface water yet are otherwise composed of similar ice surfaces (Figs. 3, 5a). However, in comparison to distributed impurities, supraglacial water has a minor impact on the mesoscale albedo pattern and explains only 15% of the albedo variability across our survey transect. Surprisingly, the supraglacial lake located at 28 km is not associated with a significant reduction of MOD10A1 albedo. This is because it is relatively narrow (260 m width) and covers just 33% of the segment, while 25% of the remaining segment consists of very bright clean ice faculae (mean *α* = 0.58): interpreted as a shoreline exposed when the lake level dropped (Fig. 5c). Hence, the low albedo of the lake water (mean *α* = 0.19) itself is offset by the brightness of the surrounding ice surface adjacent to it yielding minimal change in the net albedo (MOD10A1) signature.

Crevassing explains 12% of albedo variability and its impact is well illustrated at 3–4 km where a transition into a crevasse zone yields a significant reduction in mesoscale albedo compared to the adjacent, flatter surface (Fig. 6a, b). Crevasses enhance shortwave radiation absorption; radiative transfer modelling indicates that the presence of crevasses can double the downward energy absorbed relative to a homogeneous, flat ice surface and reduce albedo by between 0.10 and 0.25^28,29^. The amount of radiation absorbed by crevasses is determined by their size, orientation, density and whether they are water-filled. One of the most densely crevassed areas across the transect (5.9% fractional area) (Fig. 6a), with crevasse widths up to ~10 m and depths in excess of 8 m, yields an albedo reduction of ~0.06 in comparison to the segment in Fig. 6b, which has no crevassing but similar fractions of other surface types (Figs. 3, 6b). Elsewhere, at 6–7 km, smaller crevasses, with mean widths of 5 m, have a reduced impact on mesoscale albedo, lowering it by only 0.02 in comparison to the control segment in Fig. 6b. This observation is at odds with radiative transfer modelling results because the modelled crevasses were compared against a flat, clean ice surface, which is not the case here (Fig. 6b)^28^. Cathles et al.^29^ modelled crevasses with width to depth ratios similar to the crevasses we observed and found that they have a melt enhancement factor of 1.14–1.20 at solar zenith angles of 45°, which is in broad agreement with our findings.

Increases in the fractional area of cryoconite, either in large holes or fluvial deposits, are not particularly associated with mesoscale albedo reduction across the survey transect, and surprisingly, the lowest concentration of cryoconite is actually observed within the dark zone itself (Fig. 5a). Cryoconite only occupies a very small fraction of the total coverage (1.6% maximum and 0.6% mean), which can be explained by the nature of the cryoconite material itself. The thread-like, filamentous structure of cyanobacteria enables them to entangle debris and facilitate the formation of granules. These granules absorb more solar radiation and melt down into the ice until they are in radiative and thermodynamic equilibrium^30–32^. Although this mechanism means that the cryoconite hole has a very low albedo value (mean *α* = 0.10) when observed from directly above, the hole occupies a relatively small area and is effectively hidden at non-zenith solar illumination resulting in an increase in mesoscale albedo^20^. Furthermore, cryoconite holes are often covered by an ice lid, caused by the refreezing of water that has filled the hole during negative net radiation conditions^31^. While thin frozen lids may undergo partial or complete ablation during the day, their higher albedo acts to further moderate the impact of cryoconite that they cover and render the holes indistinguishable from the adjacent ice surface (much to the dismay of many a field campaigner with sodden feet).

## Discussion

The analysis presented here demonstrates that the dark zone has low fractional areas of surface water (<1.0%), cryoconite holes (<0.5%) and crevasses (<0.2%). Instead, it appears that ice containing uniformly distributed impurities draped over a relatively flat surface are the primary agent responsible for the low (MOD10A1) albedo values observed during the melt season (Fig. 5a). Near the S6 AWS, from where the UAV was launched, Stibal et al.^22^ report that samples of distributed impurities consist of an abundance of ice algae (Fig. 4a), which are characterized by a grey/brown hue due to the brown-to-purple coloured pigments surrounding the algae chloroplasts^24,33,34^. Correlations between dust content and the abundance of microbes suggest that the melt-out of particulates may provide nutrients for surface ice algae to grow^22,35^ and indirectly control the extent of dark zone^12^. Further support for this hypothesis is provided by Tedstone et al.^12^ who argue that the large interannual variability in the extent of the dark zone, and its significant reduction in 2013 and 2015, demonstrates that bare ice albedo is not a consequence of summer ablation alone^12^. Instead, positive correlations between the dark zone extent and proxies for the availability of liquid water and nutrients are interpreted as evidence that blooms of surface ice algae control bare ice albedo across the dark zone. Any increase in temperature and/or liquid water production in the presence of dust promotes further colonization of surface algae yielding an increase in pigmented biomass and net albedo reduction^24,36^. The 12% expansion of the dark zone between 2000 and 2014 in western Greenland, corresponding with an increase in mean summer air temperature of 0.13 °C per year over the same period^11^, provides further support for these ongoing processes.

While our results attest that the variation in the fractional area of supraglacial lakes, streams, crevasses and cryoconite do not significantly affect mesoscale albedo, they still play a secondary role in determining interannual and seasonal albedo variability. For example, supraglacial water may act to consolidate or distribute sediment and impurities across the ice sheet surface^37^. Moreover, a relatively small expansion in the spatial extent of surface water would have a disproportionate impact on mesoscale albedo and further amplify ablation. Melt rates at the base of supraglacial lakes and water bodies are double that of bare ice surfaces due to enhanced shortwave radiation absorption^38,39^. Atmospheric warming has been shown to increase the spatial extent and duration of ponded supraglacial water^40,41^. During years with higher summer temperatures, such as 2007, 2010 and 2012, supraglacial lakes formed earlier in the season and occupied a 40% larger area than in cooler summers^40^. It follows that increased storage of water in supraglacial lakes will play an important role in net albedo reduction across an expanding bare ice area in future.

Our analysis also demonstrates that crevasses reduce local (0.1–1 km) albedo, and hence any increase in crevasse extent will impact on mesoscale albedo patterns. Crevasses form due to localized concentration of tensile stresses which, due to highly variable subglacial conditions and longitudinal stress coupling, are spatially and temporally variable across the Greenland Ice Sheet^16,42,43^. In response to increased surface melt, GPS observations by van de Wal et al.^44^ report reduced net flow over the marginal zone of the K-transect, whereas Doyle et al.^45^ report persistent ice flow acceleration above the equilibrium line up to 140 km from the ice sheet margin in this same sector. Recent modelling^46^ and observations^46,47^ of new crevasses forming over 160 km from the western margin reveals that the ice sheet interior is also more dynamically sensitive to transient stress perturbations originating from downstream than a previous steady-state model suggests^48^. Regardless of ice dynamics, inland migration of the equilibrium line caused by atmospheric warming will drive increased bare ice extent, further exposing existing crevasses that were formerly snow and firn covered. Hence, surface crevasse extent will likely expand in future, resulting in mesoscale albedo reduction and enhanced surface absorption of incoming energy available for melt.

Finally, future spatial expansion of cryoconite does have the potential to significantly impact surface albedo. Hodson et al.^49^ showed that 53% of plot-scale (0.01–0.5 m) variation in albedo was correlated with the growth of cryoconite holes, and Chandler et al.^21^ report that the gradual seasonal reduction in albedo also correlates with an increase in cryoconite hole size and number. An increase in the extent of cryoconite holes may be caused by longer and warmer ablation seasons, which would increase the heat energy to the walls and base of the hole, leading to further melting and hole expansion^31^. On the other hand, an increase of meltwater may promote aggregation of distributed impurities and could have a surface cleaning effect, potentially raising the albedo of the surrounding bare ice^20,31^. The growth and development of cryoconite holes on mesoscale albedo is hence complex and still somewhat ambiguous.

In this study, we characterized the spatial variability of surface types across the western ablating margin of the ice sheet towards the end of the melt season when bare ice surfaces are most apparent. However, for much of the year (September to May), the ice sheet is snow-covered and it is likely that snow grain size and impurity concentration govern mesoscale albedo patterns across the K-transect and elsewhere during this period. Likewise, early melt-season albedo patterns are primarily governed by the relative proportions of snow and ice extent. Accurately determining snow melt and the timing of bare ice exposure has therefore been a priority for surface mass and energy balance models and the theoretical determinants of snow albedo and melt are relatively well established^5,50^. In contrast, few studies have investigated the albedo of the bare ice surface types that characterize the ablation zone and they have commonly been treated as temporal and spatial constants in surface melt models^51–53^.

The observed spatiotemporal variability in albedo across the ablation zone^54,55^ has motivated a new generation of surface energy balance models that assimilate spatial patterns of albedo derived from MODIS data^4,56,57^. Across our 25 km survey transect, the MODIS-derived surface albedo pattern is dominated by variations in the extent of uniformly distributed impurities, a result that contradicts previous research attributing it to an increased occurrence of supraglacial water^18^. The source, processes and drivers of distributed impurities are yet to be unequivocally established, with some studies indicating a wind-blown origin, others revealing that they are derived from melt-out of englacial dust^9–11^. Recent research has promoted the concept of bioalbedo, which argues that the melt-out and release of surface particulates and nutrients fertilizes pigmented ice surface algae, which drives albedo reduction over the duration of the melt season^12,22^. Further research though is required to determine how these factors combine to increase the spatial extent and concentration of pigmented surface algae, and their interaction with the availability of in situ and aeolian-derived nutrients, changing atmospheric forcing and enhanced ice melt and runoff.

## Methods

### MODIS albedo

Albedo patterns were determined from a MOD10A1 C6 broadband (spectral range of 300–3000 nm) albedo product from the 8 August 2014 and available from the National Snow and Ice Data Center (NSIDC)^58^. MOD10A1 is gridded in a sinusoidal map projection and has a resolution of ~500 × 500 m or 0.25 km^2^. The value of each pixel represents the best single albedo observation in the day based on cloud cover and viewing and illumination angles^59^. We estimated that MOD10A1 has a root mean square difference (RMSD) of 7.0% in comparison to albedo measured by CNR1 or CNR4 thermopile pyranometers at the PROMICE/GAP automatic weather stations KAN-L, KAN-M and KAN-U between 2009 to 2014^60^. This compares well to Stroeve et al.^59^ who estimated an RMSD of 6.7%.

### UAV platform

Aerial imagery was acquired by a fixed-wing UAV identical to that used by Ryan et al.^14,61,62^ The UAV has a 2.1 m wingspan and is powered by a 10 Ah, 16.8 V LiPo battery pack which, with a total weight of 4 kg, yields a 1 h endurance and 60 km range. The autonomous control system is based around an Arduino navigation and flight computer updated in real-time by a 10 Hz data stream comprising of a GPS, magnetometer, barometer and accelerometer. These data are logged along with a timestamp for each activation of the digital camera shutter which automatically triggers when a horizontal displacement threshold is exceeded. The UAV was hand launched on 8 August 2014 from a base camp at 67.08°N, 49.40°W, located at the site of the Institute for Marine and Atmospheric Research (IMAU), University of Utrecht S6 automatic weather station. It was pre-programmed to carry out a 25 km survey across the Kangerlussuaq sector of the western Greenland Ice Sheet (Fig. 1). The Greenland Ice Mapping Project (GIMP) digital elevation model (DEM)^63^ was used during the selection of three-dimensional waypoints to ensure the UAV maintained a constant altitude of 350 m above the surface during the autonomous sorties. On return from the sortie, the UAV was manually landed into a 10 × 5 m net.

### Digital imagery

Digital imagery was acquired by a Sony NEX-5N digital camera vertically mounted inside the front of the airframe. The camera has a 16 mm fixed focus lens (53.1 by 73.7° field of view) yielding an image footprint of ~525 × 350 m during the autonomous sortie. The width of each image is approximately similar to the pixel footprint of MODIS. The camera was preset with a fixed shutter speed of 1/1000 s, ISO 100 and F-stop of 8, and triggered every 35 m to provide a 90% forward image overlap. The relatively fast shutter speed minimizes image blur while the low ISO and F-number ensures maximum image quality where even the brightest surfaces do not saturate the image. The camera was set to record the images in RAW format, an image format that contains minimally processed data from the camera’s sensor. During the survey, ~2000 RAW images were acquired at the camera’s maximum (4912 × 3264 pixels) resolution which, once the images were corrected for barrel, or geometric, distortion, equates to a ground sampling distance of ~11 cm.

### Orthomosaic and DEM generation

The R(ed)/G(reen)/B(lue) images were used to produce an orthomosaic and DEM using Agisoft PhotoScan Pro (http://www.agisoft.com/) following the processing sequence described by Ryan et al.^60^ The images were georeferenced by providing latitude, longitude and altitude data recorded by the flight controller. The orthomosaic was produced in the software’s ‘mosaic’ mode, meaning that pixels in the centre of the images were preferentially used to provide the output pixel value. The orthomosaic and DEM were nearest neighbour resampled to a ground resolution of 15 cm and 50 cm, respectively. We divided the orthomosaic into sixty 0.25 km^2^ segments and each segment was assigned a MOD10A1 value.

### Surface classification

**Fig. 7:**
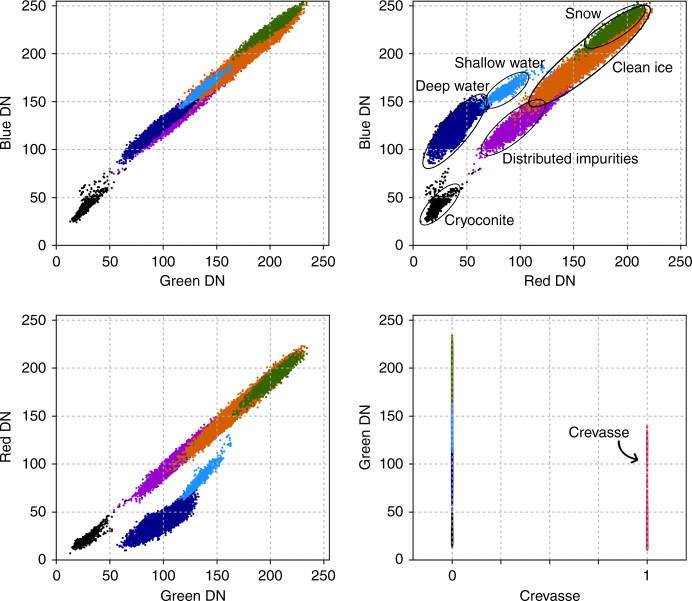
Scatter-plots showing the key attributes of the seven surface types identified in this study. DN is digital number of the Sony NEX-5N calibrated RAW image. Deep and shallow supraglacial water is easily distinguishable because it has low reflectivity in the red band and forms a unique cluster in the feature space. Snow, clean ice, ice containing distributed impurities and cryoconite form another cluster and are distinguishable because they reflect different proportions of RGB visible wavelengths

The fractional area of each surface type was calculated by dividing the number of pixels of each surface type by the total number of pixels in each orthomosaic segment. The number of pixels of each surface type was estimated using a supervised k-NN classification from the scikit-learn Python module^64^. The pixels were classified using a majority vote based on the Euclidean distance to five equally weighted nearest neighbours (Fig. 7). The k-NN was manually trained with seven distinct and visually identified surfaces found in the orthomosaic: (i) clean ice, (ii) ice containing uniformly distributed impurities, (iii) deep water, (iv) shallow water, (v) cryoconite either in holes or fluvial deposits, (vi) crevasses and (vii) snow. The training samples of the surface types were manually digitized from 10 orthomosaic segments based on RGB brightness and a layer that specified whether or not the pixel was situated within a crevasse or fracture. This roughness layer was determined by calculating the residual between the original 50 cm DEM subtracted from a 30 m Gaussian-smoothed DEM. Negative anomalies with a vertical displacement >1 m were identified as crevasses. Small cracks and fractures were detected on the basis of sharp RGB contrast, and were discriminated using an edge detector algorithm^65^. Pixels within 2 m of a linear feature were also identified as crevasses.

The efficacy of the k-NN classifier was evaluated by comparison with independently digitized surface types in three orthomosaic segments, at the centre and extreme ends of the transect. We found that 92% of the pixels were classified accurately. The k-NN classified crevasses with an accuracy of 88% and performed better for deep and shallow water (96%) than for cryoconite, ice containing distributed impurities, clean ice and snow (90%). The classification of supraglacial water is relatively accurate because water has low reflectance in the red band and forms a unique cluster in the feature space (Fig. 7). The performance of the k-NN for clean ice and ice containing distributed impurities is less accurate (90%) because these surfaces reflect the RGB visible bands in similar relative proportions and the surface classes overlap in the feature space (Fig. 7). Misclassification could also be caused by shadows, especially in segments with steep topography (eg, Fig. 6b). Shadows increase the proportions of the surface classified as ice containing distributed impurities and/or cryoconite. Accounting for shadows would reduce the proportion of ice containing distributed impurities in the crevassed zone (Fig. 6b) and subsequently increase their variation across the transect. This would make the impact of distributed impurities on the mesoscale albedo variability, and the conclusions of this study, more significant. Finally, we used PCR to explore the dominant modes of surface type variation along the transect and assess which surface type, or set of surface types, best correlate with mesoscale albedo variability, as represented by MOD10A1. Linear regression was used to yield the correlation coefficients between the principle component scores and MOD10A1 albedo.

### Estimating albedo of digital image pixels

An estimate for the albedo (*α*) of each surface type was obtained from the UAV digital imagery. An explicit description of this method can be found in Ryan et al.^14^, but we briefly summarize it here. Firstly, the RAW digital numbers of each proprietary Sony RAW image were preserved by converting to a 16-bit TIFF image using dcraw (http://cybercom.net/~dcoffin/dcraw/). A vignette correction mask was universally applied to compensate for image and lens distortion due to edge effects which were as high as 17.6% at the corners of some images. The correction mask was calculated from the mean vignette of all images acquired at nadir during the survey period. Barrel distortion was corrected using ImageMagick (http://www.imagemagick.org/), which utilized the coefficients stored in the image’s ancillary metadata also known as exchangeable image file format data.

We then corrected the images for changing illumination conditions during the survey using downward irradiance measured by a ground-based upward facing Apogee SP-110 pyranometer. To do this, images of a 25 × 25 cm Teflon white reference target were acquired every 10 min using the UAV digital camera from the ground. The relationship between the mean RGB DNs of the white reference target and the downward irradiance recorded by the upward facing pyranometer were used to construct a calibration curve using a linear least squares regression (*R*^2^ = 0.96). The ratio of reflected radiation recorded by the camera and the downward radiation estimated from the calibration curve enabled the illumination-corrected reflectance of each pixel to be defined.

Since snow and ice are non-Lambertian surfaces, a nadir measurement of reflectance underestimates albedo by between 1 and 5% in the visible band^66^. The illumination-corrected images were therefore calibrated again by multiplying the image pixel numbers by a factor calculated by dividing the mean pixel value of the illumination-corrected image by the albedo recorded by upward and downward facing Apogee SP-110 pyranometers mounted on the UAV. Ryan et al.^14^ found that albedo determined using this method has an accuracy of ±5% over ice sheet surfaces typically found in the ablation zone.

### Data availability

The MODIS (MOD10A1) albedo data are available from the National Snow and Ice Data Center (NSIDC) at http://nsidc.org/data/MOD10A1. The UAV images are archived in the PANGAEA repository: https://doi.pangaea.de/10.1594/PANGAEA.885798.
